# Effectiveness of silver diamine fluoride in indirect pulp capping in primary molars: A systematic review and meta-analysis

**DOI:** 10.1016/j.heliyon.2023.e19462

**Published:** 2023-09-07

**Authors:** Khlood Baghlaf, Asrar Ehsan Sindi, Fatimah Abdullah Almughalliq, Norah Khalid Alarifi, Rahaf Alquthami, Reema Abdullah alzahrani, Sultana Alhaid

**Affiliations:** a Pediatric Dentistry Department -King Abdulaziz University, Jeddah, Saudi Arabia; b MOH, Saudi Arabia; c Imam Abdulrahman Bin Faisal University, Saudi Arabia; d King Saud University, Riyadh, Saudi Arabia; e Umm Alqura University, Makkah, Saudi Arabia; f NYU, College of Dentistry, NY, USA

**Keywords:** indirect pulp capping, Indirect pulp treatment, Silver diamine fluoride, Primary teeth, Primary molars, Primary dentition

## Abstract

**Introduction:**

Recently, clinical trials have assessed the effectiveness of Silver Diamine Fluoride (SDF) as an indirect pulp capping material (IPC) in primary teeth. This systematic review aimed to assess the evidence presented in these trials.

**Data sources:**

A comprehensive search identified relevant studies through five electronic databases (PubMed, Scopus, ClinicalTrials.gov, ScienceDirect, and Cochrane). Search strategies were designed using the PICO model to identify all studies that investigated SDF as an IPC compared to calcium hydroxide (CaOH) or mineral trioxide aggregate (MTA). Quality assessment and Grading of Recommendations Assessment, Development, and Evaluation (GRADE) were used to assess the level of evidence.

**Study selection:**

Four clinical trials were found to be suitable for inclusion in the qualitative synthesis and three studies were included in the quantitative analysis. Three studies compared SDF with CaOH and only one study compared SDF, CaOH, and MTA. Only one randomized controlled trial (RCT) had a low risk of bias, and the non-RCT study had a moderate risk of bias. The level of evidence based on the GRADE was low. Three out of four studies showed higher clinical and radiographic outcomes with SDF than with CaOH. One non-RCT study showed that SDF resulted in the least reparative dentin at the 6-months follow-up. The meta-analysis showed a non-significant difference between the SDF and CaOH groups (P = 0.36).

**Conclusion:**

There is little evidence showing a higher clinical and radiographic outcome of SDF compared to CaOH as an IPC material in primary molars.

**Clinical significance:**

This systematic review updates pediatric dentists regarding the effectiveness of using SDF as indirect pulp caping in primary teeth.

## 1 Introduction

Dental caries is considered a significant oral disease in children [1]. It is one of the most common chronic diseases affecting children. It is formed by bioacid formation, which leads to chemical dissolution of the enamel [1].Deep carious lesions are a challenge in the treatment of primary teeth [2]. If there are no signs or symptoms of pulp involvement, indirect pulp capping (IPC) or indirect pulp treatment (IPT) is advised [3]. The main goal of this technique is to arrest caries progression and preserve pulp vitality by forming a dentin-like matrix (tertiary dentin) as part of the repair of the dentin-pulp complex [4].

IPC agents have been used in the management of deep carious lesions in vital teeth. Calcium hydroxide (CaOH), introduced by Hermann in 1921 as a root canal filling [5], is the gold standard for pulp capping [6]. It has had long-term clinical success as a pulp capping agent for follow-up of up to 10 years [7]. However, it possesses unfavorable properties such as improper seal and poor adhesive quality, and the so-called “tunnel defects” in reparative dentin has been shown to form underneath CaOH [8].

Mineral trioxide aggregate (MTA) has been investigated for various applications since the early 1990s [9]. It was introduced by Torabinajed and showed less pulp inflammation and more predicted hard-tissue formation than calcium hydroxide (Coll et al., 2017). MTA is a pulp sealing material composed of a mixture of tricalcium silicate, dicalcium silicate, tetracalcium aluminoferrite, and calcium sulfate dehydrate with the addition of bismuth oxide in a 4:1 ratio [6].

Silver diamine fluoride (SDF) is a chemical component composed of 45,000 ppm fluoride mixed with silver [10]. The mechanism of action of SDF is the inhibition of biofilm formation; moreover, it permits enamel to reduce solubility and acts as an antimicrobial agent. The FDA has approved SDF as a liner material and dentin desensitizer [11], and its success has been proven in arresting active carious lesions in primary teeth [12,13]. It is also suitable for uncooperative patients: after applying a protective layer to the lip and skin of the patient, the affected tooth is air dried and the area is isolated with a cotton roll; one drop of SDF is applied to the microbrush and added directly to the carious lesions; the excess material is then removed and the tooth is air dried again [14]. The main drawback of using SDF material is the black staining caused by the oxidization of silver, which makes it inconvenient for some parents, especially when applied on the anterior teeth [15].

To the best of our knowledge, no systematic review has assessed the effectiveness of SDF as IPC material in primary teeth. In this systematic review, we aimed to compare the clinical and radiographic effectiveness of SDF to CaOH as IPC material in primary teeth. Moreover, this systematic review aimed to assess the evidence that compares SDF to MTA as IPC material in primary teeth, in terms of clinical and radiological results.

### 1.1 Research questions

This systematic review addressed two research questions.

1 Is the clinical and radiographic success of SDF as an IPC material in primary teeth comparable to that of calcium hydroxide?
2 Is the clinical and radiographic success of SDF as an IPC material in primary teeth comparable to that of MTA?

## 2 Methods

### 2.1 Study selection

The systematic review was conducted in accordance with the Preferred Reporting Items for Systematic Reviews and Meta-Analyses (PRISMA) standard [16]. The study was registered in the International Prospective Register of Systematic Review (PROSPERO) database (CRD42022355263) in September 2022. Studies included in this review were selected following the PICOS elements (Centre for Reviews and Dissemination, 2009).

• Participants: healthy children aged 4–10 years with deep caries in primary teeth indicated for IPC.
• Intervention/exposure: using silver diamine fluoride (SDF) on deep caries in primary teeth.
• Comparison: with a control group using MTA and/or CaOH on deep caries in primary teeth.
• Outcomes: clinical and radiographic success rate.
• Study design: all clinical studies including randomized clinical trials (RCTs) and non-RCTs.

The excluded studies did not include children or studies that assessed the success of SDF in IPC of permanent teeth. Studies that assessed SDF as IPC and compared it to materials other than calcium hydroxide and MTA were excluded. In addition, all case reports and clinical studies with samples <10 teeth were excluded.

### 2.2 | information source and search strategy

A comprehensive search identified relevant studies in five electronic databases (PubMed, Scopus, ClinicalTrials.gov, ScienceDirect, and Cochrane). Other sources were also utilized in this search, including hand searches in high-impact factor journals and Google Scholar. There was no restriction on the time of publication (up to September 2022) and the search was limited to English-language articles. Search strategies were designed to identify all studies that investigated the clinical and radiographic outcomes of SDF as an IPC material compared to CaOH or MTA. The following keywords were used during the search: indirect pulp capping, indirect pulp treatment, silver diamine fluoride, primary teeth, primary molars, and primary dentition.

### 2.3 | Data extraction

The following information was extracted from the studies into the data extraction forms: author and year, study setting, research methodology (study design, number and age of children, number of teeth, type of teeth, presence of SDF, CaOH, MTA, and sample size in each group), follow-up period(s), and clinical and radiographic outcomes. Using a data extraction sheet, reviewers R. A. and R. A. A. independently collected data from the selected studies. In this systematic review, we defined treatment success based on the accomplishment of specific clinical and radiographic criteria. The clinical criteria were as follows: no pain, swelling, abscess, pain on percussion, and mobility. The radiographic criteria were the width of the tertiary dentine measured using software or standardized radiographs and the absence of radiolucency.

### 2.4 | Quality appraisal

To assess the risk of bias in each included RCT, two reviewers, N. A. and S. A., used cochrane's risk of bias tool [17]. The Newcastle Ottawa Scale was used to assess the methodological quality of non-RCTs [18]. The meta-analysis included studies of moderate and high methodological quality (higher than five stars). In case of any discrepancy between the two reviewers, a third reviewer, K. B., handled the discussion to reach an agreement. The level of agreement between the two authors was evaluated using a kappa value.

The Grading of Recommendations Assessment, Development, and Evaluation (GRADE) Working Group criteria were used to assess the quality of each research question [19]. Two reviewers A.S. and S.A. were assigned to assess the GRADE and a third reviewer K.B. handled any disagreement. Based on the design restrictions, risk bias, precision, directness, and consistency of the results, the GRADE method was able to grade the overall quality of evidence. The GRADE analysis also categorized the quality of evidence as high, moderate, low, or very low. Any limitations in the quality of the study or inconsistencies in the results caused a marked decrease in the quality grade of the evidence.

### 2.5 Meta analysis

RevMan software was used to conduct the meta-analysis (version 5; 1, Nordic Cochrane Center, Cochrane Collaboration, 2001). Cochran's Q-test, with a statistically significant P value of 0.1, was used to check for heterogeneity in the studies. Pooled studies had low to moderate heterogeneity (>25%–75%). Statistical significance was set at p < 0.05. Quantitative synthesis requires a minimum of two investigations. A random-effects model was conducted in the presence of two or more studies using the same assessment tool.

## 3 Results

### 3.1 Study selection

Our Search retrieved 1680 articles obtained from five different databases: ScienceDirect (n = 588), PubMed (n = 226), Cochrane (n = 619), Scopus (n = 214), and ClinicalTrials.gov (n = 33). Other sources were also utilized in this search, including manual searches in high-impact-factor journals and Google Scholar. Using these additional sources, we were able to retrieve additional 1130 articles. The search was conducted until August 2022.

**Fig. 1:**
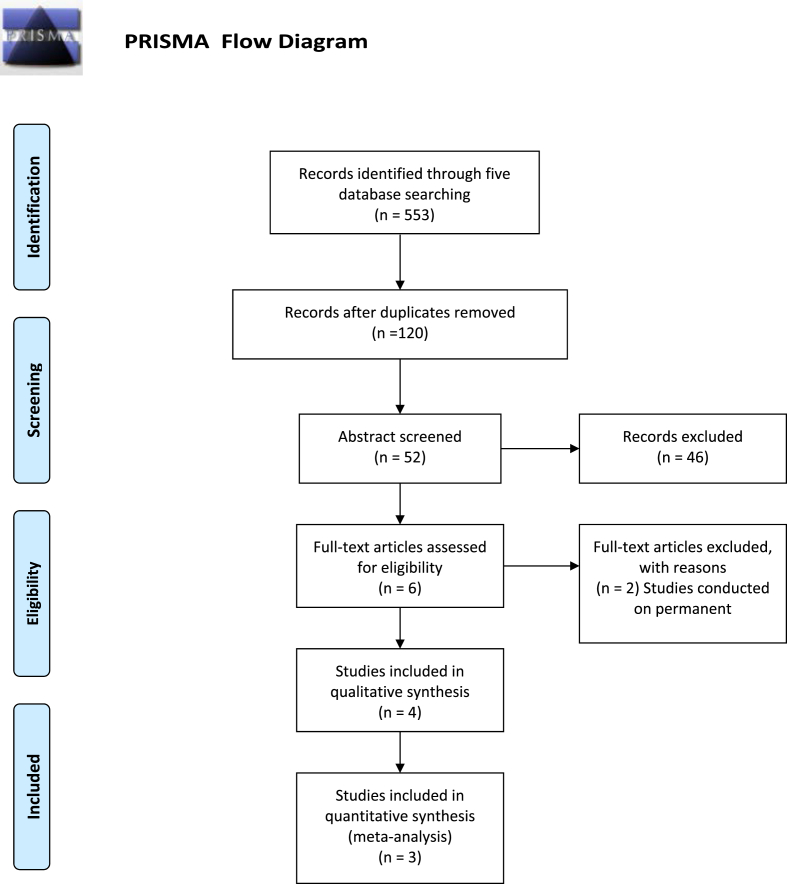
The PRISMA flow chart of the systematic review.

After removing duplicates, only 1**20 articles remained for the abstract screening stage.** Only six studies remained after title screening. After full-text screening, only four articles were found to be suitable for inclusion in the qualitative synthesis. Only two studies were pooled in the meta-analysis after they were assessed for quality (Fig. 1).

### 3.2 Study characteristics

Table 1, Table 2 summarize the key characteristics of the studies included in the qualitative synthesis. All four studies were conducted in India. The children were aged from 4 to 10 years. Three RCTs [[20], [21], [22]] and one non-RCT study [23] were included in the systematic review. The sample sizes ranged from 75 teeth (25 in each group) to 34 teeth (17 in each group). Three studies compared SDF with CaOH [20,22,23]and only one study included SDF, CaOH, and MTA [21]. The follow-up period ranged from 1 to 12 months. The RCT by Shafi et al., 2022 [20] included diluted SDF, which is one drop of SDF mixed with nine drops of distilled water giving a 1:10 dilution and used light cure CaOH.

**Table 1:** Characteristics table of the studies that compared SDF to calcium hydroxide (CAOH) in indirect pulp capping of primary teeth.

Author-date	Site	Study design	Number and Age of children	Number and type of teeth	Method of assessment	SDF Clinical/radiographical	CAOH Clinical/radiographical	Final restoration
Shafi et al., 2022	India	Randomized clinical trial	56 children Mean age 5.7	56 primary molars 28 dilute SDF 28 light cure CaOH	Criteria of success: the presence of an intact tooth with normal periodontium, and intact lamina dura, Criteria for failure included: the presence of pain, sensitivity to percussion, intraoral or extraoral sinus/abscess/swelling, widening of periodontal ligament space, internal resorption of the root, external resorption of the root, radiolucency in furcal area.	The success of SDF was 96% at the end of 12 months. The difference between the two groups was not statistically significant **(p>0.05**). However, dilute SDF can be a potential treatment option as an indirect pulp capping material in primary molars with deep caries lesions due to the high success rate.	The success of light cure calcium hydroxide was 91.6% at the end of 12 months.	The indirect pulp treatment was followed by glass ionomer cement restoration and all primary molars received stainless steel crowns.
Divyashree - 2021	India	Randomized controlled in vivo study	75 children 6–9 years	75 teeth Primary molars 25 SDF 25 CAOH 25 MTA	Clinical sign and symptoms: No pain, no sensitivity to percussion, no swelling and/or fistula, no pathologic tooth mobility, retention of the restoration (Marginal integrity) Radiographic: formation of reparative dentine, no radiolucency in periapical or furcation are, no widening of periodontal ligament space, no external or internal resorption	The success of 38% SDF was evaluated using PA and Corel draw software The reparative dentin was around 0.0076 mm by the end of 6 months from baseline. This gives a statistically insignificant **P- Value of 0.83.** SDF showed the lowest amount of reparative dentin formation among all three groups.	The success of CAOH was evaluated using PA and Corel draw software The reparative dentine formed was around 0.1534 mm by the end of 6 months from baseline.	All teeth were restored with Resin Modified Glass Ionomer Cement RMGIC to ensure a proper seal
Patil et al., 2021		Randomized control trial	Number of children not mentioned Age 4–7 years	50 teeth Primary molar 25 SDF 25 CAOH	Clinical success criteria: absence of spontaneous night-time pain, sensitivity to pressure or any stimulus, mobility, abscess, sinus, fistula and swelling of periodontal tissue in each follow-up examinations. Radiographic success criteria: increase in the remaining dentin thickness, absence of interradicular radiolucency, periodontal ligament space thickening and signs of pathological root resorption in each follow-up radiograph.	Clinical and radiographic success 3-month follow-up:24 (96%) Clinical and radiographic Success 6-month follow-up: 24 (96%) No significant difference between the outcomes of the two materials	Clinical and radiographic success 3-month follow-up:23 (92%) Clinical and radiographic success 6-month follow-up: 22 (88%)	Permanent restoration for SDF group was then done using resin modified glass ionomer cement. Permanent restoration for CAOH group was using resin modified glass ionomer cement.
Shah et al., 2020	India	Non-randomized clinical trial	27 children 4–10 years	34 teeth Primary anterior and posterior teeth 18 SDF 16 CAOH	Clinical success criteria: presence or absence of post-treatment signs or symptoms such as sensitivity, pain or swelling Radiographic success criteria: evaluated for the presence or absence of any pathologic changes.	Clinical and radiographic success at 1 month was found to be 100%. There is no significant difference between the two groups.	Clinical and radiographic success at 1 month was found to be 93.75%	All teeth were restored with GIC Fuji IX

**Table 2:** Characteristics of the studies compared SDF to MTA in indirect pulp capping of primary teeth.

Author-date	site	study design	Number and Age of children	Number and type of teeth	Method of assessment	SDF Clinical/radiographical	MTA Clinical/radiographical	Final restoration
Divyashree et al., - 2021	India	randomized controlled in vivo study	75 children 6–9 years of both sexes	75 teeth Primary molar 25 SDF 25 CAOH 25 MTA	Clinical success criteria: no pain, no sensitivity to percussion, no swelling and/or fistula, no pathologic tooth mobility, retention of the restoration (Marginal integrity) Radiographic success criteria: formation of reparative dentine, no radiolucency in periapical or furcation area, no widening of periodontal ligament space, no external or internal resorption	The success of 38% SDF was evaluated using PA and Corel draw software. The reparative dentine formed was around 0.0076 mm by the end of 6 months from baseline. This gives a statistically significant **P- Value of 0.001** SDF showed the lowest amount of reparative dentin formation among all three groups.	The success of MTA was evaluated using PA and Corel draw software. The reparative dentine formed was around 0.1166 mm by the end of 6 months from baseline	All teeth were restored with Resin Modified Glass Ionomer Cement RMGIC to ensure a proper seal

### 3.3 Quality and risk of bias

**Fig. 2:**
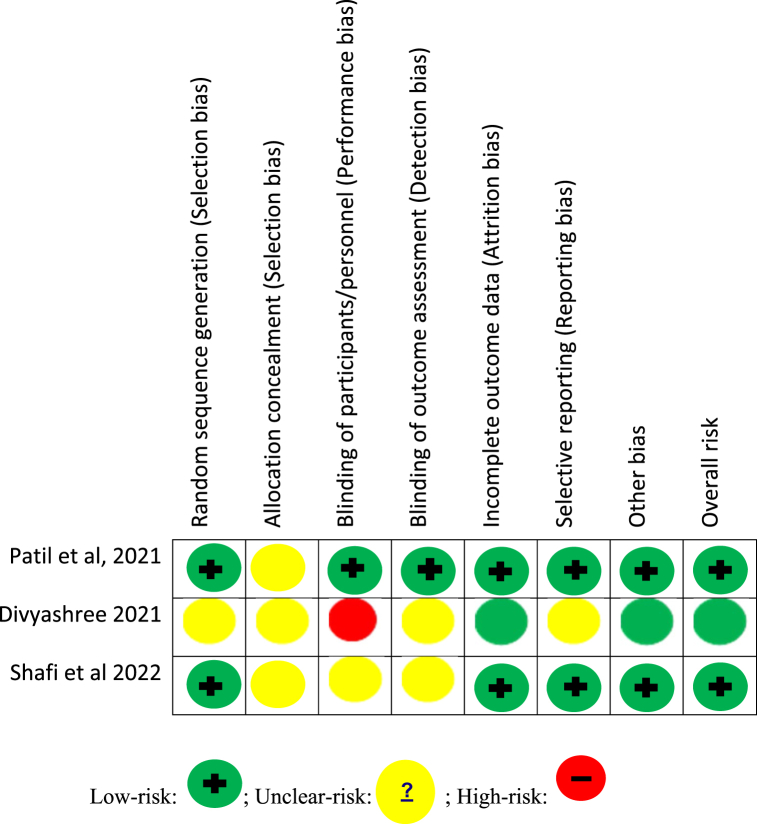
Cochrane's risk of bias assessment.

**Fig. 3:**
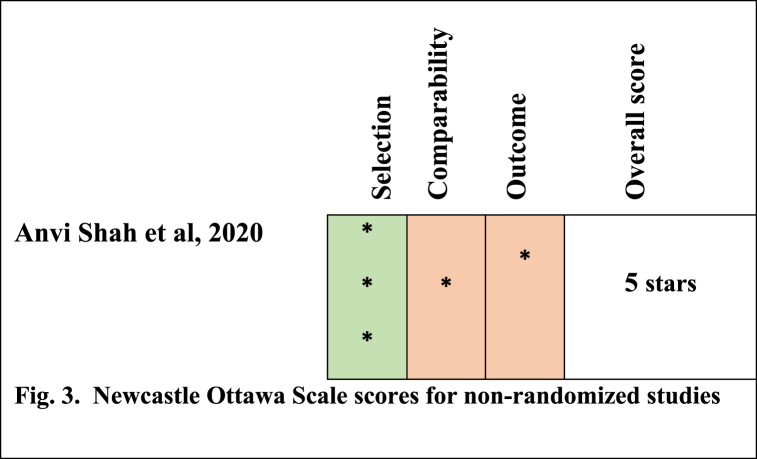
The Newcastle Ottawa Scale for non-RCT studies.

Three randomized clinical trials [[20], [21], [22]] were included for Cochrane risk of bias assessment and were assessed by two authors. The inter-rater agreement for the evaluation of the Cochrane risk of bias was very good (kappa score = 82). One RCT was found to have a low risk of bias [22] with randomization and blinding of both participants and outcome assessment. The other RCTs [20,21] were found to have an unclear risk of bias (Fig. 2). The non-RCT [23] was found to have a moderate risk of bias (five stars) (Fig. 3). The inter-rater agreement for the evaluation of the Newcastle-Ottawa scale was good (kappa score = 80).

### 3.4 Level of evidence (GRADE)

GRADE rating for only one research question (Is the clinical and radiographic success of SDF as an IPC material in primary teeth comparable to that of CaOH?) was determined according to the type of study (RCTs). A GRADE profile table was created (Appendix 1) with three RCTs [[20], [21], [22]]. Downgrading was performed for serious risks of bias, inconsistency, and imprecision. However, the level of evidence was low.

### 3.5 SDF as indirect pulp capping material

Three of the four included studies showed favorable results for SDF when used as an IPC material in primary teeth. Shafi et al., 2022 showed 96% clinical and radiographic success in the diluted SDF group and 91.6% in light cure CaOH at the end of 12 months. One RCT by Patil et al., 2021 [22] showed 96% clinical and radiographic outcomes of SDF at the 6-months follow-up compared with 88% success with CaOH. An RCT by Divyashree, 2021 [21] showed a higher amount of reparative dentin in the CaOH group, followed by the MTA group. In this study, SDF formed the least amount of reparative dentin. There was a highly statistically significant difference between the three groups at the end of 6 months. A non-RCT study by Shah et al., 2020 [23] showed 100% clinical and radiographic success using SDF as IPC material compared to 93.75% using Dycal at the 1-month follow-up.

### 3.6 Meta analysis and sensitivity test

**Fig. 4:**
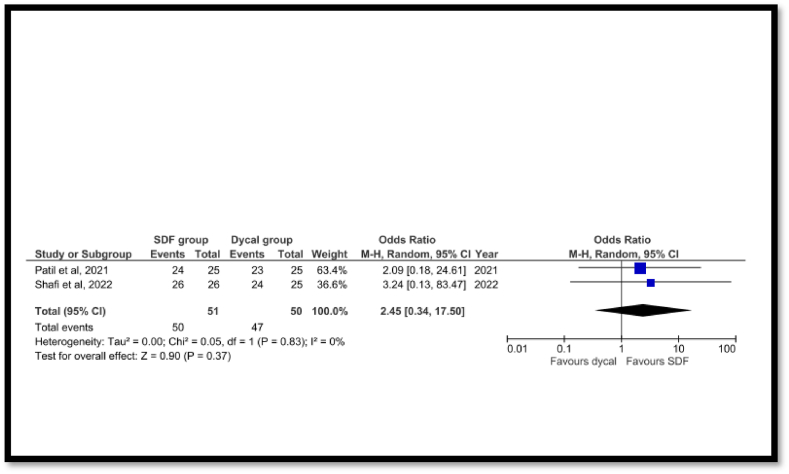
Forest plot showing the clinical and radiographic success of SDF compared to calcium hydroxide (CAOH) for 3–6 months follow-up.

Fig. 4 shows the clinical and radiographic success of SDF compared with CaOH in IPC in primary teeth at the 3–6 months follow-up. Only two RCTs [20,22] were included in this meta-analysis, and the findings showed a non-significant difference between the two groups with an overall effect of z = 0.90 (p = 0.37). Heterogeneity was zero (I^2^ = 0%).

A sensitivity analysis was conducted between the different RCTs and non-RCTs to demonstrate the stability and reliability of the meta-analysis. The meta-analysis (Appendix 2) showed consistent results of no significant difference between the SDF and CaOH groups, with results favoring SDF, and the effect size was higher z = 1.14 (p = 0.25) with zero heterogeneity (I^2^ = 0%).

## 4 Discussion

This systematic review aimed to assess the evidence comparing the effectiveness of SDF as IPC in primary teeth with MTA and CaOH. Based on a previous systematic review [24], SDF is effective in arresting caries in primary teeth. Over the years, SDF has shown great success rates due to its superior properties as a cavity liner due to its ability to inhibit caries progression and its effect on tooth desensitization in both primary and permanent teeth [25,26].

In pediatric dentistry, the current direction of treatment is toward preventive and less invasive nonsurgical procedures [27]. According to the AAPD guidelines, IPC has shown greater success rate regardless of the materials used [28]. The common materials used in IPC for primary teeth are MTA, CaOH, and glass ionomer cement. In recent studies, a smart dentin replacement is introduced in IPC of primary molars [29]. Biodentine is a bioactive calcium silicate-based material, that preserve and repair the remaining pulp tissue [30].

None of the studies included in this systematic review [[21], [22], [23]] investigated several applications of SDF when used as IPC. According to studies that investigated SDF in arresting caries, there was no significant difference between applying SDF once a year and fluoride several times a year [31]. However, future clinical trials should be directed to compare the effectiveness of several applications of SDF when used in IPC of primary teeth.

Only two clinical trials included in this systematic review described the remaining dentin thickness according to their inclusion criteria [21,22]. Both clinical trials included only carious lesions involving more than two-thirds of the thickness of the dentine, approximating the pulp with an intact lamina dura. An in vivo study compared the response of the pulp to different materials, including SDF, in second premolars [32]. This study found that when the remaining dentin thickness ranges between 0.25 and 0.5 mm, a higher response of tertiary dentin deposit is observed. However, when the remaining dentin thickness >0.5 mm or <0.25 mm, it shows a lesser response of tertiary dentin deposition. This is because it allows the byproducts from the restorative material to reach the pulp and show cytotoxic effects, leading to the death of preexisting odontoblasts [32,33].

SDF contains approximately 253,900 ppm silver and 44,800 ppm fluoride ions [34]. Two concentrations are available for SDF in the market (38% and 12% SDF) and previous studies found that 38% SDF was more effective in arresting caries [35,36].Only one RCT used diluted SDF FAgamin and 38% SDF manufactured by Tedequim SRL, which can be applied to deep caries at a dilution ratio of 1:10 with distilled water [20]. All the other three clinical trials used non-diluted 38% SDF. The study by Divyashree, 2021 [21] used 38% SDF manufactured by Elevate Oral Care, whereas Patil et al., 2021 [22] used 38% SDF manufactured by Tedequim SRL. The non-RCT by Saha et al., 2020 [23] reported the use of 38% SDF; however, they did not mention the manufacturing company.

A systematic review that investigated the response of SDF on pulp [37] found that topical application is effective in preventing caries in primary teeth and may have a significant impact on reducing the burden of untreated dental disease in children. In this systematic review, only one study on the occurrence of pulp necrosis was assessed in an individual trial [38]. None of the included studies reported pulp necrosis as a failure of SDF. Shafi et al., 2022 reported that only one case at the 6-months follow-up failed in the diluted SDF group and this clinical failure was due to pain [20]. The study referred to this failure as an inaccurate baseline pulp diagnosis or a pulp reaction to the pulp capping material [20].

Meta-analysis showed a non-significant difference between the SDF and Dycal as IPC materials in primary molars, with results favoring the SDF group. However, these results should be interpreted with caution because the level of evidence was low. A sensitivity test was also performed, including RCTs and non-RCT studies, which showed the same findings. however, the overall effect was higher. In this systematic review, only the study in Ref. [21] that compared SDF and MTA as IPC materials in primary teeth. Therefore, a meta-analysis was not performed.

One of the limitations of this systematic review is the limited number of primary studies exploring the effectiveness of SDF in IPC of primary teeth with long follow-up periods. Other limitations including the potential effect of confounders including patient age, lesion size, SDF concentration, and final restorative material. Only one RCT reported clinical and radiographic success at 1-year [20]. The lack of high-quality RCTs examining the effectiveness of SDF in IPC emphasizes the need for high-quality, well-designed RCTs. Future RCTs should investigate several applications of SDF when used as IPC in primary teeth and compare the success of the diluted form.

## 5 Conclusion

Three out of four clinical trials showed a higher clinical and radiographic outcome with SDF in comparison to CaOH; however, the level of evidence was rated as low. One non-RCT study showed a significant difference between SDF and other groups (MTA and Dycal), and SDF resulted in the least reparative dentin at the 6-months follow-up. Further controlled RCTs with a long follow-up period are needed to investigate the clinical and radiographic success of SDF as an IPC in primary molars.

## Author contribution statement

All authors have made substantial contributions to all of the following: conceived and designed the experiments; performed the experiments;, analyzed and interpreted the data; contributed reagents, materials, analysis tools or data & wrote the paper.

## Declaration of competing interest

The authors declare that they have no known competing financial interests or personal relationships that could have appeared to influence the work reported in this paper.
