# Supplementary material for: Effectiveness of silver diamine fluoride in indirect pulp capping in primary molars: A systematic review and meta-analysis
Source: Heliyon. 2023 Sep 7;9(9):e19462. doi: 10.1016/j.heliyon.2023.e19462 (PMC10558611; doi:10.1016/j.heliyon.2023.e19462)
Supplement: Multimedia component 1 [file mmc1.docx]

**Appendix 1. Grade Profile Tables**

**Table S1 QUESTION: Is the clinical and radiographic success of SDF as indirect pulp capping material in primary molars comparable to calcium hydroxide?**

**Settings: General child population (with primary teeth)**

| **Quality assessment** | | | | | | | **No of patients** | | | **Effect** | **Quality** |
| --- | --- | --- | --- | --- | --- | --- | --- | --- | --- | --- | --- |
| **No of studies** | **Design** | **Risk of bias** | **Inconsistency** | **Indirectness** | **Imprecision** | **Other considerations** | **Teeth treated with SDF** | | **Control (CAOH)** |  |  |
| \| **Dental caries (cross-sectional studies); measured with standard indices; better indicated by lower values)** \| \| --- \| | | | | | | | | | |  |  |
| 3 | Randomized clinical trials ^1^ | serious risk of bias^2^ | \| serious inconsistency^3^ \| \| --- \| | no serious indirectness^4^ | serious imprecision^5^ | \| confounding ^6^ \| \| --- \| | 78 | 78^7^ | | \| pooled^8^ \| \| --- \| | \| ⨁◯◯◯  Low   \|  \|  \| \| --- \| --- \| \| \| --- \| --- \| --- \| |

^1^ Three non-randomized clinical trials (Shafi et al 2022, Divyashree . 2021, Patil et al, 2021) investigated the clinical and radiographical effectiveness of silver diamine fluoride to calcium hydroxide an indirect pulp capping material. One of the three studies showed statistically significant difference in the amount of reparative dentine favouring Calcium hydroxide group, the other two studies found higher clinical and radiographical success rate favouring SDF group. Meta-analysis was not possible due to the variability in the outcome measure.

^2^ In Shafi et al 2022 & Divyashree . 2021 there was lack of blinding and lack of allocation concealment while in Patil et al, 2021 there was blinding and allocation concellment; therefore, there was a downgrading for risk of bias.

^3^ There is evidence of inconsistency. Therefore, downgrading was done for this inconsistency. Divyashree et al, 2021 showed the reparative dentine formed in SDF group was around 0.0076 mm by the end of 6 months from baseline and in calcium hydroxide the reparative dentine formed was around 0.1534mm by the end of 6 months from baseline. This gives a statistically insignificant P- Value of 0.83., Patil et al, 2021 showed Clinical and radiographic success at 6 months was found to be 96% in SDF group and 88% in calcium hydroxide group. Shafi et al 2022 showed 96% clinical and radiographic success in SDF and 91.6% in light cure calcium hydroxide at the end of 12 months.

^4^ Data were not downgraded for indirectness because all randomized trials were conducted in primary molars in children. All three studies were conducted in India.

^5^ Downgrading for imprecision because if the effect estimate comes from only three small studies with few events.

^6^ No downgrading due to the plausible confounding was done , Patil et al, 2021 only selected cooperative children with primary molars but Divyashree . 2021 study wasn’t controlled for the other confounding factors such as patient cooperation, isolation of the tooth and type of the teeth (upper or lower molars) .

^7^ Total number of teeth from the 3 randomized clinical trials.

^8^ pooling due to variability in the outcome measures only two RCTs were able to pooled z=0.90 , p=0.37 with low heterogeneity I_2_.=0% Divyashree et al, 2021 assessed the reparative dentine formed in mm while Patil et al, 2021 and Shafi et al 2020 only presented the clinical and radiographic success for each group.


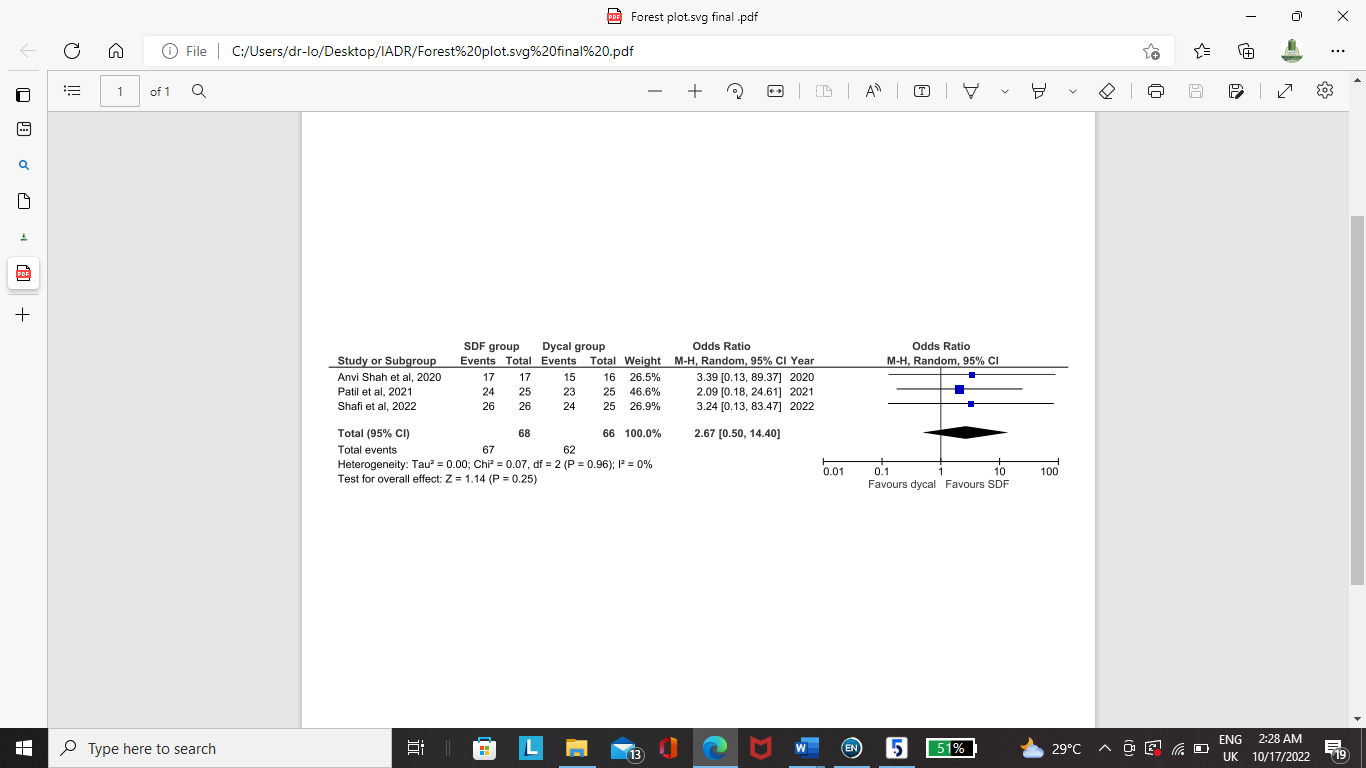


Appendix 2: Forest plot showing the clinical and radiographic success of SDF compared to CAOH for 3-6 months follow-up.
